# What is the best available evidence for treatment of first metatarsophalangeal joint osteoarthritis?

**DOI:** 10.1186/1757-1146-4-S1-P61

**Published:** 2011-05-20

**Authors:** Gerard V Zammit, Hylton B Menz, Shannon E Munteanu, Karl B Landorf, Mark F Gilheany

**Affiliations:** 1 Musculoskeletal Research Centre, Faculty of Health Sciences, La Trobe University, Bundoora, Victoria 3086, Australia; 2 Department of Podiatry, Faculty of Health Sciences, La Trobe University, Bundoora, Victoria 3086, Australia; 3 Australasian College of Podiatric Surgeons, East Melbourne, Australia

## Background

Osteoarthritis (OA) affecting of the first metatarsophalangeal joint (MTPJ), is a common and painful condition. Although several different treatments have been suggested throughout the literature, few have been adequately evaluated. Therefore, the aim of this study was to conduct a Cochrane review in order to determine the efficacy of treatments available for first MTPJ OA.

## Methods

Randomised controlled trials, quasi-randomised trials, or controlled clinical trials that assessed treatment outcomes for first MTPJ OA were searched for across several electronic databases (to the 14th January 2010) (CENTRAL; MEDLINE; EMBASE; CINAHL; and PEDro). Participants of any age or gender with OA of the first MTPJ defined either radiographically or clinically were included in this review.

## Results

Only one trial satisfactorily fulfilled the inclusion criteria and was included in this review. This trial evaluated the effectiveness of two physical therapy programs in 20 individuals with OA of the first MTPJ. Assessment outcomes included pain levels, first MTPJ range of motion and plantarflexion strength of the hallux. Mean differences at 4 weeks follow up were 3.80 points [95% CI: 2.74 to 4.86] for self reported pain, 28.30 degrees [95% CI: 21.37 to 35.23] for first MTPJ range of motion and 2.80 kilograms [95% CI; 2.13 to 3.47] for muscle strength. Although differences in outcomes between treatment and control groups were reported, the risk of bias was high. The trial failed to employ appropriate randomisation or adequate allocation concealment, used a relatively small sample and incorporated a short follow up (4 weeks). No adverse reactions were reported.

## Conclusions

Only one quasi-randomised controlled trial for first MTPJ OA was found. The reviewed trial presented a high risk of bias, limiting the conclusions that could be drawn from the presented data. The inclusion of only one trial indicates the need for more robust randomised controlled trials to determine the efficacy of interventions for this condition.

